# Evaluating the Validity of Simplified Chinese Version of LIWC in Detecting Psychological Expressions in Short Texts on Social Network Services

**DOI:** 10.1371/journal.pone.0157947

**Published:** 2016-06-20

**Authors:** Nan Zhao, Dongdong Jiao, Shuotian Bai, Tingshao Zhu

**Affiliations:** 1 Key Laboratory of Behavioral Science, Institute of Psychology, Chinese Academy of Sciences, Beijing, China; 2 National Computer System Engineering Research Institute of China, Beijing, China; 3 School of Information Engineering, Hubei University of Economics, Wuhan, Hubei, China; Hangzhou Normal University, CHINA

## Abstract

The increasing need of automated analyzing web texts especially the short texts on Social Network Services (SNS) brings new demands of computerized text analysis instruments. The psychometric properties are the basis of the extensive use of these instruments such as the Linguistic Inquiry and Word Count (LIWC). For this study, Sina Weibo statuses were analyzed via rater coding and Simplified Chinese version of LIWC (SCLIWC), in order to evaluate the validity of SCLIWC in detecting psychological expressions in Weibo statuses (n = 60) and in identifying the psychological meaning of a single Weibo status (n = 11). Significant correlations between human ratings and SCLIWC scores and the high sensitivities of capturing single statuses with certain expressions identified by raters, proved the validity of SCLIWC in detecting psychological expressions. The results also suggested that, the efficiency of SCLIWC in detecting psychological expressions of SNS short texts could be higher if using status count scoring method, rather than the word count method as the common usage of LIWC. However, SCLIWC may not perform well in identifying the psychological meaning of a single piece of SNS short text because of its over-identification of target expressions. This study provided primary evidence of validity of SCLIWC, as well as the proper way of using it efficiently on SNS short texts.

## Introduction

People’s daily language profoundly reflects their psychological worlds [1]. Today as the explosion of online textual data coming from people’s daily life naturally and spontaneously, the need to interpret the psychological aspects coded by language in online communication [2] and the need for valid computer-based methods of rapidly analyzing texts have been increasingly highlighted. While there were a range of general purpose computerized text analysis programs in psychology having been developed, such as General Inquirer [3], Wordnet [4] and Opinion Finder [5], the Linguistic Inquiry and Word Count (LIWC) [6] is now often the preferred automated text analysis method in psychology, and an important choice of natural language processing in computer sciences. LIWC was developed in early 1990s [7] to map psychological and linguistic dimensions of written expression, and then it was keeping updated. Composed by a text processing program and the dictionaries, LIWC could calculate a percentage of words falling into 80 psychologically or linguistically meaningful categories. These categories cover several important psychological aspects of an individual, including emotion, cognition, social contact and personal concerns. Another significant benefit of LIWC is that as a truly transparent text analysis method, the manipulation of output variables is totally visible to users and it allows users to extend the lexicons or even add new categories to meet their needs.

In past 20 years, LIWC has been used in hundreds of studies exploring the relationships between psychological processes and the word categories in daily language. The language features depicted by LIWC word categories have been found to reflect user’s attentional focus [8], emotionality [9], social status and hierarchy [10], social coordination and group processes [11], Deception [12], close relationships [13], cognitive styles [14], mental health status [15] and other individual differences [16]. Tausczik and Pennebaker’s review [17] has provided a detailed list of studies of this approach. LIWC was also used in computer science as a natural language processing tool to extract computable features from online textual data, especially in the recent boom of social media research. With the LIWC word categories as parts of the feature sets for computational prediction models, scientists could predict users’ personality [18–20], personal values [21], tie strength [22], mental health status [23–24], subjective well-being [24–25], and even political election result [26] based on the textual data of social medias and other sources.

No matter for which approach above, the validity of LIWC is a crucial issue. When the words of a category are used, does that mean the user do express the meaning as that category defined? The answer of this question could largely determine the interpretation of the relationship between word categories and psychological processes, as well as the effectiveness of the word categories as feature sets. Considering the large amount of work using LIWC as a tool, independent studies regarding its psychometric properties are quite few, especially for the validity of categories other than emotional expression. A direct evidence of LIWC validity was the comparison between human ratings and LIWC variables. Pennebaker and Francis [7] required judges to rate essays written by college students along 12 dimensions of LIWC, using a 7-point unipolar scale, and the validity was represented by the correlations between judges’ ratings of the category with the LIWC variable. Their results showed that for categories of emotion processes and some cognitive processes, there were medium to high correlations between human ratings and LIWC variables. Similar method was also used to provide evidence of LIWC validity in some other reports [27–28]. Another way of comparison was reported by Bantum and Owen [29], where raters reviewed each word and coded it into a specific emotion category or as being absent of emotion, and then compared with LIWC variable. The LIWC emotion lexicons were found to be quite effective in detecting emotional expressions. This method used the signal-detection indices to qualify the validity of the lexicon and was also implemented in recent study [30]. In addition, other validity data, such as the comparison of the LIWC value pattern among different studies, or the comparisons of LIWC values among different types of texts, were also reported [27].

Although there has been some evidence that LIWC was valid in processing many types of English texts, more work is still needed as LIWC being more widely used. First, while LIWC was translated into other languages, the validity data on that language would be a necessary basis for the applications after translating. Second, the validity of LIWC on texts of Social Network Service (SNS) is worthy to be evaluated. Compared to the essays and forum discussions used to test LIWC validity in previous studies, today SNS is often inundated with short texts, such as tweets and Weibo statuses. While facing to a bunch of independent short texts with various topics, could LIWC play as well as on those long texts with a single topic? This is a very valuable question since there is a huge demand to processing SNS texts with instruments like LIWC.

To meet the needs of processing Simplified Chinese texts, especially the web texts, Gao et al. [31] developed a Simplified Chinese version of Linguistic Inquiry and Word Count (SCLIWC) based on LIWC [28] and the Traditional Chinese version of LIWC (CLIWC) [32]. The SCLIWC was first translated from CLIWC and then each word was checked for its category through the same method used to develop LIWC [28]. Since there is no wildcard in Chinese, in order to improve the word capture rate of SCLIWC lexicon on today’s masses Chinese, the high frequency words extracted from Chinese SNS were added into the lexicon according to the LIWC categories [31]. However, for the application of SCLIWC, the validity data is still needed. In this study, we analyzed Simplified Chinese web text with the aim to answer two questions: How accurate the detection of psychological expressions was on different web texts by SCLIWC; and how to use SCLIWC in a more efficient way to detect psychological expressions on SNS short texts. To accomplish these aims, we employed the method comparing human ratings and LIWC variables as in several previous studies, and used three different types of web text: Sina Weibo statuses as the SNS short texts, Renren blogs as the SNS long texts, and news comments as the traditional web texts.

## Study 1

This study was aimed at evaluating the validity of SCLIWC for identification of psychological expression in web text. Three different web texts: Weibo statuses, Renren blogs, and news comments, were processed by both SCLIWC and human raters. The validities of SCLIWC with different scoring methods on Weibo statuses of different time spans were assessed and compared in order to reveal the more effective way of identification.

### Method

#### Participants and Materials

Sina Weibo is a popular social media site in China which is similar to Twitter. The Weibo statuses (like tweets) from April 1, 2012 to April 30, 2012 of 60 Weibo users (30 males and 30 females, based on the gender information they filled in their Weibo profiles) were used in this study. These users were randomly selected from our active Weibo user pool [33], who met the following requirements:

1. During Jun1 2012 to Jun30 2012, each user posted 90–110 valid statuses.
2. During Jun1 2012 to Jun7 2012 (the first week), each user posted 20–26 valid statuses.
3. On Jun1 2012, each user posted 3–4 valid statuses.

Here the valid status means those whose word count was larger than 0 after deleting links, reposted content (“//@username:” or marked in the “retweeted_status” field of downloaded data object through Sina Weibo API), mentions (“@username”) and emotion icons. We downloaded the texts of these 60 users’ 5,931 valid statuses in April 2012. A Weibo status may include links, mentions, emotion icons, pictures, audio or video clips, and reposted contents. Since the aim of this study was to evaluate the validity of SCLIWC in processing texts, the content beyond the LIWC processing scope such as links, emotion icons, pictures, audio or video clips were removed. We would like to focus on the expression of Weibo users, while the mentions were others’ username and the repost contents were usually mixed with non-personal expression such as advertisements and news, so the mentions and reposted contents were also dropped, living only the original text expressed by the Weibo user of each status. The average text length of such a “cleaned” status was 25.2 Chinese characters with a range from 1 to 140 (the upper limit of one Sina Weibo status) characters. In this sample male users posted almost equal number of statues as females did (98.7 vs. 99.0), but their status length was a little shorter than females’ (23.8 vs. 26.6).These cleaned statuses were used for rater coding, and for SCLIWC scoring the mentions were further removed.

Renren is a popular social networking site in China which is similar to Facebook, and there are many users posting blogs through their Renren account. Sixty Renren blogs of 60 users (30 males, 30 females) were selected in the current study, which were all about the experiences, thinking and feelings of the writers, with 1552.1 characters (SD = 562.4) on average for each. The 60 news comments, published during 2012–2014, were selected in some China’s mainstream media websites (such as Xinhua and Sina), whose topics covered current politics, economy and social affairs, with 1603.9 characters (SD = 444.1) on average.

The Weibo statuses and Renren blogs were used in this study in the context of participants’ electronically informed consent. As detailedly described in Li et al.’s study [33], the participants were recruited online through an informed-consent web page with two buttons “I agree” and “I disagree”. Only if one clicks “I agree” to provide his/her informed consent to participant in this study, could we download and use his/her Weibo statuses or Renren blogs. This research plan was approved by the Institutional Review Board of Institute of Psychology, Chinese Academy of Sciences.

#### Rater coding

Eighteen categories of SCLIWC were selected to be assessed in this study, covering personal pronouns (First Person), social processes (Family, Friends), affective processes (Positive Emotion, Negative Emotion, Anxiety, Anger, Sadness), cognitive processes (Insight, Causation, Discrepancy, Tentative), biological processes (Biological Processes) and personal concerns (Work, Achievement, Leisure, Money, Death). The category First Person here was created by merging the SCLIWC categories First Person Singular and First Person Plural. The selected categories of personal pronouns, social processes, affective process, cognitive process and biological processes were assessed by human judges in previous studies [28] and most of them were found to be relevant to some psychological outcomes [17]. The objects and events people care about may also be an important reflection of their mind, so we added some categories of personal concerns in our list.

For each given text, the raters in our study made the decision of whether, or how much, it was characterized by each of the 18 categories. The definitions of these categories referred to Pennebaker and Francis’s study [7] and Bantum and Owen’s study [29]. Our raters were required to evaluate the meaning of the whole content rather than detect certain words. For example, if a Weibo status obviously states the author’s performances or feelings, it would be characterized by the category *First Person*, no matter whether there was personal pronoun in this status or not; Similarly, those statuses using the Chinese character “death” in a colloquial way to express a strong attitude rather than discussing real death, would not be identified regarding to the category *Death*. We also excluded those statements using emotional words just to express some preference, e.g., “Nan likes icecream of green tea flavor”, while giving the ratings on affective processes, in order to identify those “real” emotional expressions.

We trained 3 graduate students of psychology institute as the raters of this study. Then they independently coded all the texts while being blind to the SCLIWC scoring results. Cleaned Weibo statuses were presented in a single work sheet for each Weibo user, without any supplementary information except the time it was posted. For each Weibo users, the raters made the judgments in sequence on how much the first day/first week/whole month’s Weibo statuses could be characterized by each category on a scale from “1” (“none”) to “7” (“quite a lot of”). For each Renren blog and news commentary, similar judgments were made for the whole article. The average of the 3 raters’ rating scores was the final human rating scores used in further analysis. The reliability of the 3 raters was measured using Cronbach alphas, and this index was found to be acceptably high for each category on each of the three text types, between .78 (Discrepancy on Weibo statuses) and .99 (Sad on news comments).

#### SCLIWC scoring

Since there is no space between words as the word boundary in Chinese as in English, all the texts used in this study were firstly segmented into single words through Language Technology Platform (LTP) [34]. Then, the SCLIWC was conducted to count words of different categories in the texts. For the sets of Weibo statuses, Renren blogs and news comments, the word count of each category (the SCLIWC word count score) was directly calculated. For the single Weibo status, if there was one or more word of a SCLIWC category appearing in a status, this status would be labeled as the same category. For example, “I feel depressive today” was labeled as a *sad* statu*s* because the word “depressive” was in the SCLIWC *sad* category. Then the number of statuses in each category (the SCLIWC status count score) for each Weibo users was calculated. These two SCLIWC scores as well as the human ratings were put into SPSS 15.0 for further analysis.

### Results and Discussion

#### The validity of SCLIWC in detecting psychological expressions in Weibo statuses, Renren blogs, and news comments

As the usual way to analyzing texts using LIWC lexicon, the proportion of the word count of each category in the total text word number was firstly calculated and compared with human ratings. Table 1 shows the percentage of total words identified for Weibo statuses, Renren blogs and news comments in our study. In this table the inspected SCLIWC categories are briefly divided into 4 groups: self and others (mentioning person), affective processes, cognitive processes, and concerned contents (mentioning objects except person). The word percentages of most of these categories are similar to the means listed by LIWC’s authors for results of analyses of multiple texts written under different instructions [28]. Only for the category First Person, the word percentage is much lower than Pennebaker et al.’s results [28], which may reflect the characteristic of Chinese utterance. Meanwhile, the word percentages on different text types show discrepancies consistent to the features of the type: news comments were expressing opinions in an objective perspective and a rational manner, so they use much less first person and more causation words than Weibo and Renren; for the contents, Weibo and Renren were more personalized while news comments focused on public topics like economy and policies, so news comments mentioned less words of Biological process and Leisure, but much more words of Work, Achievement and Money.

**Table 1:** The percentage of total words detected by the SCLIWC categories on Weibo statuses, Renren blogs and news comments.

	self and others	affective processes	cognitive processes	concerned contents
Weibo	Ist Per. 2.74(1.96)	Pos. Emo. 3.40(1.62)	Insight 1.87(1.06)	Bio. 3.40(1.81)
	Family .48(.38)	Neg. Emo. 1.69(.98)	Caus. 1.08(.70)	Work 2.72(1.68)
	Friend .19(.15)	Anxiety .23(.21)	Disc. 2.58(1.71)	Achieve 1.30(.82)
		Anger .49(.50)	Tent. 2.16(1.23)	Leisure 2.23(1.22)
		Sad .35(.29)		Money .69(.51)
				Death .31(.35)
Renren	Ist Per. 3.44(1.73)	Pos. Emo. 2.54(1.04)	Insight 2.52(1.05)	Bio 2.23(1.42)
	Family .33(.50)	Neg. Emo. 1.62(.81)	Caus. 1.33(.64)	Work 2.73(1.82)
	Friend .18(.22)	Anxiety .23(.19)	Disc. 2.40(.98)	Achieve 1.57(1.19)
		Anger .32(.25)	Tent. 2.72(1.13)	Leisure 1.46(1.16)
		Sad .37(.42)		Money .49(.50)
				Death .13(.22)
Media	Ist Per. .44(.47)	Pos. Emo. 2.11(1.34)	Insight 2.77(.96)	Bio .94(1.55)
	Family .16(.39)	Neg. Emo. 1.16(.82)	Caus. 2.11(.81)	Work 8.71(3.79)
	Friend .13(.54)	Anxiety .20(.24)	Disc. 1.93(.69)	Achieve 3.86(2.01)
		Anger .35(.39)	Tent. 1.86(.83)	Leisure .97(1.57)
		Sad .15(.19)		Money 1.50(1.76)
				Death .17(.59)

The standard deviation (SD) was presented in the parentheses following the mean percentage. The results of Weibo statuses were calculated from all the valid Weibo statuses collected of each Weibo user in our study.

To examine the validity of SCLIWC in detecting psychological expressions on Weibo statues, Renren blogs and news comments, we conducted Pearson Correlation analysis between the SCLIWC word count scores and the corresponding human ratings as Pennebaker and Francis [7] did (Table 2). For the categories about self and others, SCLIWC scores were significantly correlated with human ratings, but the correlations were small or medium, expect for the two categories, Family and Friend, high correlations were achieved on news comments. For affective processes, the correlations between SCLIWC scores and human ratings on three text types were close, and the small to medium degree of correlation was consistent with previous studies [7, 27]. The correlations between SCLIWC scores and human ratings were not significant for most categories of cognitive progresses on Weibo statuses and news comments, but were significant and achieved medium for Insight, Causation and Tentativeness on Renren blogs. Most of the correlations of concerned contents were significant except for Death category on Weibo statuses, and most of them achieved moderate, or even high on Renren blogs and news comments.

**Table 2:** correlations between human ratings and SCLIWC word count scores on Weibo statuses, Renren blogs and news comments.

	self and others	affective processes	cognitive processes	concerned contents
Weibo	Ist Per. .37**	Pos. Emo. .33*	Insight .25	Bio. .44**
	Family .37**	Neg. Emo. .46*	Caus. .29	Work .47**
	Friend .29*	Anxiety .57**	Disc. .18	Achieve .35**
		Anger .41**	Tent. .17	Leisure .41**
		Sad .35**		Money .43**
				Death .22
Renren	Ist Per. .37**	Pos. Emo. .37**	Insight .59**	Bio. .70**
	Family .53**	Neg. Emo. .44**	Caus. .43**	Work .59**
	Friend .28*	Anxiety .36**	Disc. -.05	Achieve .38**
		Anger .27*	Tent. .62**	Leisure .57**
		Sad .46**		Money .84**
				Death .73**
Media	Ist Per. .27*	Pos. Emo. .37**	Insight .12	Bio. .70**
	Family .71**	Neg. Emo. .50**	Caus. .15	Work .39**
	Friend .74**	Anxiety .46**	Disc. .15	Achieve .53**
		Anger .26*	Tent. .57**	Leisure .85**
		Sad .38**		Money .80**
				Death .83**

*p < 0.05

**p < 0.01.

The current results show the validity of the SCLIWC word count score in detecting psychological expressions in Weibo statuses, Renren blogs, and news comments. The similarity of the word percentage profile of selected categories in our study with Pennebaker et al.’s [28] results, as well as the discrepancies of the profiles among different text types, are confirming the construct validity of SCLIWC. Moreover, the significant correlations between SCLIWC scores and human ratings are direct evidence of the concurrent validity of SCLIWC. As shown in Table 2, the validities of different categories on different text types are variant: the validities of Family and Friend are higher on news comments than on Weibo and Renren; the validities of the categories of cognitive process are quite low on Weibo statuses and news comments (except Tentativeness), but much better on Renren blogs (except Discrepancy); for those categories about concerned contents, the validities on Renren and media are higher than on Weibo. However, in general the validity of SCLIWC in the current study achieves the level of the validities of LIWC in previous studies [7, 27].

#### Comparing the validities of SCLIWC with different counting methods on Weibo statuses of different time spans

The results above confirms the validity of SCLIWC in detecting psychological expressions on a considerable amount of Weibo statuses (a month’s statuses), whose total word number reached the level of a typical essay (like a blog or a media commentary) on the internet. However, as the length of text drops, the possibility of errors made by the psychological semantic dictionary may increase. To examine the validity of SCLIWC on texts with less words, we conducted Person Correlation Analysis on human ratings and SCLIWC scores on Weibo statuses in a day, a week and a month (Table 3). In general, the correlations between human ratings and SCLIWC scores changed not much among different quantities of Weibo statuses. The number of categories with significant correlation rose a little as the number of statuses increasing from a day’s to a week’s, and from a week’s to a month’s. It seems that SCLIWC did perform better in detecting psychological expressions in analyzing texts with larger number of words, nevertheless it also showed high validity in many categories on the texts as short as a day’s Weibo statuses (3–4 statuses) in current study.

**Table 3:** Correlations between human ratings and SCLIWC scores (word count/status count) on Weibo statuses of different time spans.

	self and others	affective processes	cognitive processes	concerned contents
day	Ist Per. .63**/.67**	Pos. Emo. .26*/.51**	Insight .19/.31*	Bio. .57**/.55**
	Family .49**/.43**	Neg. Emo. .40**/.66**	Caus. .16/.30*	Work .24/.27*
	Friend .01/.09	Anxiety .54**/.71**	Disc. -.07/.16	Achieve .22/.14
		Anger .51**/.44**	Tent. .01/.17	Leisure .17/.37**
		Sad .40**/.53**		Money .61**/.74**
				Death .61**/.65**
week	Ist Per. .55**/.62**	Pos. Emo. .59**/.63**	Insight .46**/.48**	Bio. .56**/.60**
	Family .42**/.32*	Neg. Emo. .39**/.60**	Caus. .30*/.31*	Work .42**/.44*
	Friend .16/.17	Anxiety .39**/.39**	Disc. -.05/.08	Achieve .03/.21
		Anger .30*/.41**	Tent. .15/.26*	Leisure .38**/.46**
		Sad .52**/.56**		Money .57**/.63**
				Death .55**/.58**
month	Ist Per. .37**/.57**	Pos. Emo. .33*/.51**	Insight .25/.47**	Bio. .44**/.55**
	Family .37**/.55**	Neg. Emo. .46*/.50**	Caus. .29*/.59**	Work .47**/.49**
	Friend .29*/.25‘	Anxiety .57**/.43**	Disc. .18/.47**	Achieve .35**/.37**
		Anger .41**/.50**	Tent. .17/.37**	Leisure .41**/.46**
		Sad .35**/.51**		Money .43**/.45**
				Death .22/.42**

The pairs of data in bold mark the condition that the correlation was significant and the coefficient became higher when using status count as the SCLIWC scoring method.

‘*p* < 0.10

*p < 0.05

**p < 0.01.

Besides the scoring method of word count, which is the most common way in the use of psychological semantic dictionaries, status count could be another available scoring method in analyzing a group of Weibo statuses using SCLIWC. We also calculate the correlation coefficients between human ratings and the status count scores of each categories on a day’s, a week’s and a month’s Weibo statuses. As shown in Table 3, for most of the categories in which there was significant correlation between human ratings and SCLIWC scores, the coefficients became higher when using the status count scoring method, especially on the categories of cognitive processes. The consistency of the changing trends of correlation coefficients in all the four groups of SCLIWC categories implied that, when analyzing Weibo statuses the scoring method of status count may show better effect than word count, and this finding was true for different amount of Weibo statuses. When evaluating a month’s Weibo statuses, the correlations between human ratings and SCLIWC scores were significant or marginally significant in all the selected categories in our study while using status count method, and most of them achieved medium correlation, which confirmed that the dictionary SCLIWC does be a valid tool in detecting psychological expressions in Weibo statuses, if used in a proper way.

## Study 2

On social medias such as Sina Weibo, a status was the natural unit expressing a complete thought, as well as the unit of interpreting the expressions by social media users. In Study 1, the outstanding performance of SCLIWC status count score in detecting psychological expressions in Weibo statuses raised a further question: whether this method could be used to make judgments on the psychological meanings of a single Weibo status? If a single status could be classified as certain SCLIWC categories automatically and accurately based on its psychological meaning, the scope of application of SCLIWC would be further expanded. To answer this question, we conducted Study 2, which was aimed at evaluating the validity of SCLIWC for identification of the psychological meaning of a single Weibo status.

With reference to Bantum and Owen’s method [29], we used signal-detection theory [35] and the signal-detection indices to quantify the accuracy of SCLIWC identification. For our purpose to estimate whether a Weibo status with a word of a SCLIWC category does expressing the meaning of that category, a signal is the expression of the psychological meaning of a certain category, and noise is the lack of such expression. Four signal-detection indices sensitivity, specificity, positive predictive value and negative predictive value, were used in this study. Sensitivity was the probability that a status that is actually representative of the psychological expression of a SCLIWC category would be identified by SCLIWC as belonging the same category. Specificity was the probability that a status not expressing the meaning of a SCLIWC category would be identified by SCLIWC as not belonging the same category. Positive predictive value was the probability that a status characterized by SCLIWC as expressing the meaning of a category is truly representative of the meaning of that category, and negative predictive value was the probability that a status characterized by SCLIWC as not being indicative of a category is, in fact, absent of the meaning of that category.

### Method

#### Participants and Materials

Eleven (6 males and 5 females) of the 60 Weibo users in Study 1 were selected as the sample of Study 2, through simple random sampling, conducted on males and females separately. Every participant in Study 1 was given a number and eleven of them were picked out by random lottery without replacement. Each of these users posted 100.9 valid Weibo statuses with 30.0 characters on average in Jun 2012, and these texts were used in Study 2 for being coded status by status.

#### Rater coding and SCLIWC scoring

The 3 raters in Study 1 continued to do the coding in Study 2. With the 11 users’ Weibo statuses presented in the same way as in Study 1, the raters independently made the judgment that whether each single status could be characterized as each SCLIWC category or not. The coding rules were also the same as in Study 1, with the purpose of identifying the real meaning of the status rather than detecting any specific words. On each category, each status was judged by 3 raters and if there was any disagreement among them, the majority vote would be the final judgment. Interrater reliability was tested between each pair among the 3 raters, on their judgments of each category, and the kappa scores were quite high (0.64–0.90), showing that there were substantial reliability among different raters.

The SCLIWC scoring procedure was similar to the status labeling process in calculating status count score in Study 1: if there was one or more word of a SCLIWC category appearing in a status, this status would be labeled as the same category.

### Results and Discussion

To estimate the validity of SCLIWC for identification of the psychological meaning of a single Weibo status, we calculated four signal-detection indices using the data of rater coding and SCLIWC scoring: (a) *sensitivity*—the proportion of Weibo statuses identified by raters as being indicative of each category that were labeled by SCLIWC as the same category; (b) *specificity*—the proportion of Weibo statuses identified by raters as being not indicative of each category that were also labeled by SCLIWC as not being associated with the same category; (c) *positive predictive value*—the probability that a status labeled by SCLIWC as being indicative of each category was in agreement with rater codings of the same category; (d) *negative predictive value*—the probability that a status labeled by SCLIWC as being not indicative of each category agreed with raters’ judgment that the status was not associated with the same category. The sample means of the four indices on each category were shown in Table 4.

**Table 4:** The mean sensitivity, specificity, positive predictive value and negative predictive value of SCLIWC in single Weibo status scoring (N = 11).

*SCLIWC categories*	*sensitivity*	*specificity*	*positive predictive value*	*negative predictive value*
Self-references	.63(.14)	.89(.10)	.78(.22)	.76(.12)
Family	.40(.27)	.97(.03)	.32(.28)	.98(.02)
Friend	.21(.31)	.99(.01)	.56(.39)	.92(.06)
Positive emotion	.84(.19)	.69(.08)	.25(.20)	.96(.05)
Negative emotion	.65(.14)	.83(.07)	.40(.12)	.93(.04)
Anxiety	.81(.23)	.97(.01)	.38(.22)	.99(.01)
Anger	.54(.27)	.95(.03)	.41(.30)	.96(.04)
Sad	.49(.28)	.95(.05)	.34(.29)	.98(.01)
Insight	.59(.27)	.76(.10)	.19(.21)	.91(.19)
Causation	.87(.25)	.86(.08)	.16(.13)	.99(.01)
Discrepancy	.45(.31)	.67(.12)	.05(.03)	.96(.04)
Tentativeness	.65(.39)	.72(.10)	.02(.03)	.99(.01)
Biological processes	.83(.13)	.77(.09)	.54(.15)	.93(.07)
Work	.71(.19)	.82(.09)	.38(.19)	.95(.04)
Achievement	.62(.24)	.84(.07)	.20(.13)	.97(.02)
Leisure	.63(.15)	.86(.08)	.56(.16)	.88(.10)
Money	.77(.15)	.97(.03)	.64(.22)	.99(.01)
Death	.71(.41)	.97(.02)	.39(.41)	.995(.01)

The standard deviation of each mean in this table was shown in parenthesis.

As in Table 4, the validity of SCLIWC for identification of the psychological meaning of a status showed great variability on different indices, and for different categories. SCLIWC sensitivity was relatively good for positive emotion (.84), anxiety (.81), causation (.87), biological processes (.83), work (.71), money (.77) and death (.71), but not ideal for other categories in our study. SCLIWC specificity was higher than 0.70 for most of these categories except positive emotion (.69) and discrepancy (.67). The positive predictive value was generally poor and with considerable variability between categories (.02–.78). Only for self-references, this index was high (.78), which means 78% of statuses identified by SCLIWC as mentioning the author him/herself, were thought by raters as the expressions which did talk about the status author. Generally, the negative predictive value was very high for most of the categories except self-references (.76), which means 24% of statuses identified by SCLIWC as not mentioning the author him/herself, were in fact talking about the status author.

## General Discussion

### The validity of SCLIWC for detection of psychological expression in different web texts

Our first question, that the accuracy of detecting psychological expressions on different web texts by SCLIWC, was answered by the results of our two studies: SCLIWC was valid for many LIWC categories on SNS short texts (Weibo statuses), SNS long texts (Renren blogs), and traditional web texts (news comments). In Study 1, through correlation analysis between human ratings and SCLIWC variables of word count as those did in previous studies [7,27], we found significant correlations with the coefficients of generally the same level in those studies. If using status count scoring method in calculating SCLIWC variables on Weibo statuses, the correlation coefficients could be even higher. In Study 2, the evaluating based on signal-detection theory showed high sensitivity of SCLIWC for several categories and high specificity as well as negative predictive value for most categories. The validities on different LIWC categories were of large differences, as in Pennebaker et al.’s reports [28].

The difference on both content and style of the three Web text types were also reflected in our results, in both the word percentage profiles as well as the correlations between human ratings and SCLIWC variables. The discrepancies of word percentage profiles were consistent with our common sense about these text types, while the discrepancies of the correlation coefficients may provide some tips in application, that there exists some difference of SCLIWC validity on different types of Web text. For example, the accuracies of detecting expressions of affective processes using SCLIWC on Weibo statuses, Renren blogs and news comments were quite close, while the lexicon seemed to perform better in detecting concerned contents in Renren blogs and news comments.

### How to use SCLIWC to detect psychological expression in SNS short web texts more efficiently

Our second question, that how to use SCLIWC in a more efficient way to detect psychological expressions on SNS short text, was explored in two aspects: the amount of texts and the scoring method. The previous studies regarding LIWC validity usually used written materials with a substantial amount of words, such as essays of personal writing [7] or messages in online-based support groups [27,29]. Although it may not be formally discussed before, it is easy to understand that the instruments based on lexicons, such as LIWC, could perform better when the words in the material reach or exceed a certain amount. We found that SCLIWC could efficiently detect psychological expressions (medium to high correlations with human ratings) of several categories, even when the material limited to one day’s Weibo statuses (about 75–100 words on average), and as the amount of statuses increasing to a week’s and a month’s, SCLIWC could be valid on more categories.

Besides counting words, the usual way of processing texts with LIWC, counting statuses could be another option when processing a set of Weibo statuses. As in our results, the status count score generally had higher correlations with human ratings on a day/week/month’s Weibo statuses, than the word count score had. Especially for the results on a month’s statuses, the correlations between status count score and human ratings were significant on all selected categories, which indicated that the status count scoring method was a quite efficient processing method while using SCLIWC to detect psychological expressions from a set of SNS short texts. There is an important difference between a set of SNS short texts and most materials processed by LIWC in previous studies, such as personal writing essays, newspaper articles, blogs, and online-based supporting group dialogues: each piece of SNS short text is usually of a unique topic and a set of them would include many disparate topics, while other materials with similar word count are usually around one central topic. Compared to word count method, which purely uses the number of words to represent the amount of certain psychological expression, the status count method focuses on how many topics in the set relevant to certain psychological expression. When we use human ratings as the golden standard, it seems that this topic-based counting method conforms better to the cognition of human raters, and may be a preferred method to processing sets of SNS short texts through lexical instruments like LIWC.

### The validity of SCLIWC for identification of the psychological meaning of a Weibo status

Although there were significant correlations between SCLIWC status count scores and human ratings for all the selected categories, the results of using SCLIWC to make judgments on the psychological meaning of a single Weibo status do not look that good. A major problem was the poor positive predictive value. For example, this value of Sad was .34, which means about 66% statuses judged by SCLIWC as expressing sad mood actually not doing that in the view of human raters. In other words, the meaning judgments made by SCLIWC on a single status included much false alarm. This is an inherent defect of such language-processing instruments based on lexicons, as also reported in previous study [29], since these instruments equate the existence of a certain word with the expression of a certain meaning ignoring context. While using SCLIWC to identify the Weibo statuses expressing sad mood, we have to equate the appearance of a word of Sad category with sad mood expression of the author, so the sentences like “I met my depressive neighbor today” would be labeled as a sad mood expression because of the word “depressive”. The positive predictive value of Sad category reflects the proportion of such expressions in all the daily expressions with a word of Sad category. It is an important parameter for the application of SCLIWC since it defines the ability boundary of SCLIWC as a language processing tool.

The sensitivity of SCLIWC was relatively high for several categories, which means on these categories, more than 70% of the statuses expressing certain meanings could be identified by SCLIWC. For the categories for which we could find previous reports, the sensitivities were similar to (Positive Emotion and Anxiety) or lower than (Negative Emotion, Anger and Sadness) previous results [29]. These results indicated that for many categories, we could express such meanings without using any word in the lexicon of this category. This phenomenon was quite common in both English and Chinese, and may be even more in Chinese. Considering the low positive predictive value, we could conclude that through SCLIWC, large proportion of Weibo statuses expressing meanings of many selected categories could be covered, but the validity of SCLIWC to identify the psychological meaning of a single Weibo status was not ideal.

### Limitations and for future applications

It is appropriate to highlight some possible limitations in the sample of the current study. First, our sample was fairly small, especially in Study 2. Although we strictly followed the process of random sampling and included a large number of Weibo statuses in our analysis, we cannot completely exclude the possibility that the small group might have particular styles of writing Weibo statuses. Another noteworthy point was that we could only achieve limited demographic information of the Weibo users and Renren users in our sample, since many users did not provide some personal information, such as the birth date, on public SNS. Although based on the current situation of Sina Weibo and Renren, it was almost certain that the Weibo users in our sample were young adults, and the Renren users were college students, we were not able to position our sample to a certain age group more accurately. Additionally, there are other SNSs in China besides Sina Weibo and Renren, and the generalization of our findings should be considered cautiously.

This study proved the validity of SCLIWC in detecting psychological expressions in SNS short texts, while the usage of this tool would probably be in a much wider context. First, besides original texts, a Weibo status may also include links, mentions, emotion icons, pictures, audio or video clips, and reposted contents. Although SCLIWC showed its validity on interpreting textural expression of the Weibo users, which could be a most direct and explicit information of their thoughts, attitudes and emotions, a comprehensive understanding of a Weibo status still need integration of multimode. Second, Weibo (and other SNS) provides information not limited to statuses. A more ecological data mining of Weibo should use joint matrix of different aspects including users’ profiles crawled by the network spider [36], social factors, visual contents [37], and textural contents. SCLIWC could be an effective tool extracting features relevant to psychological characters from SNS textural contents, rather than independently making judgments on SNS users’ thoughts, attitudes and emotions. In future study, the SCLIWC scores, as a set of features extracted from textural content, should be a part of joint matrix served in data mining on SNS on different time granularities.

## Conclusion

The present study provides some preliminary evidence for the validity of SLIWC as an instrument for analyzing the psychological aspects of SNS short texts. The base rates of words detection, the correlations between SCLIWC scores and human ratings, and the signal-detection indices of identification accuracy proved the validity of SCLIWC from different perspectives, and suggested the method and boundary for a proper, efficient usage: 1). SCLIWC could be used in detecting the psychological expressions of the selected categories in our study, and the validity was various for different categories; 2). SCLIWC could perform better in detecting psychological expressions if using status count scoring method on a larger amount of SNS shot texts; 3). SCLIWC could NOT accurately identify the psychological meaning of a single piece of SNS short text.

As a pilot study focusing on the validity of the Simplified Chinese version of LIWC on web text, our findings not only provided culture-specific empirical data about the validity of LIWC, but also shed light on the more general issue of the LIWC usage in processing the SNS data consisting of short texts with various topics, which are now tending to inundate our cyber space. Text analysis instruments, such as LIWC, would play more significant role in research and application in this big data era, and it would be quite worthwhile to conduct deeper research, on how to use and improve these instruments to better serve the need of online data mining.

## Supporting Information

S1 Dataset The dataset of this study. (RAR)
